# Platelet-derived growth factor-BB (PDGF-BB): pathophysiological roles and therapeutic implications in aging-related diseases

**DOI:** 10.3389/fcell.2026.1926050

**Published:** 2026-09-22

**Authors:** Miao Zheng, Kai Huang, Yuanshi She, Jun Qian, Feng Zhang, Qiaocheng Zhai

**Affiliations:** 1 Department of Orthopaedics, The Second Affiliated Hospital of Soochow University, Suzhou, China; 2 Orthopaedic Institute, Wuxi Ninth People’s Hospital Affiliated to Soochow University, Wuxi, China; 3 Department of Orthopedic Surgery, The Affiliated Suzhou Hospital of Nanjing Medical University, Suzhou Municipal Hospital, Suzhou, China; 4 Division of Orthopaedics, The Quzhou Affiliated Hospital of Wenzhou Medical University, Quzhou People’s Hospital, Quzhou, China; 5 Quzhou Key Laboratory of Aging Biology and Longevity Medicine, Quzhou Affiliated Hospital of Wenzhou Medical University, Quzhou People’s Hospital, Quzhou, China

**Keywords:** aging, cellular senescence, inter-organ crosstalk, PDGF-BB, signaling pathways

## Abstract

Platelet-derived growth factor-BB (PDGF-BB), a core functional isoform of the PDGF family, serves as a central regulator of cell proliferation, migration, differentiation, and tissue remodeling. Extensive preclinical and clinical evidence indicates that PDGF-BB expression exhibits profound spatiotemporal heterogeneity during natural aging and across various pathological conditions, including cardiovascular, neurodegenerative, and musculoskeletal diseases, as well as malignancies. Importantly, these dynamic expression patterns are intrinsically linked to disease progression and distinct aging phenotypes. Mechanistically, PDGF-BB initiates complex intracellular signaling cascades—such as the ERK, JNK, PI3K-AKT, and mTOR pathways—via specific engagement with PDGFRα/β dimers. Through these networks, PDGF-BB orchestrates intercellular communication and maintains microenvironmental homeostasis in a highly dose-dependent and cell-type-specific manner. Consequently, pharmacological interventions using small molecules, natural products, and biological agents have demonstrated efficacy in modulating PDGF-BB expression and its downstream signaling, presenting promising therapeutic targets for aging-related disorders and associated tissue damage. This review systematically synthesizes the heterogeneous expression profiles of PDGF-BB across natural aging and diverse disease contexts. We delineate the molecular mechanisms underlying its role in driving senescence and pathological progression, and critically evaluate the translational potential and current limitations of PDGF-BB-targeted therapies. By comprehensively mapping the biological network of PDGF-BB, this review aims to provide a conceptual framework for future basic research and clinical translation.

## 1 Introduction

The platelet-derived growth factor family comprises four homologous polypeptide subunits—PDGF-A, -B, -C, and -D—which assemble via homo- or heterodimerization into five biologically active dimeric isoforms: PDGF-AA, -AB, -BB, -CC, and -DD (Fredriksson et al., 2004). The corresponding PDGF receptors, predominantly PDGFRα and PDGFRβ, are widely expressed on the surfaces of diverse cell types, including vascular smooth muscle cells (VSMCs), fibroblasts, osteoblasts, pericytes, and neural progenitor cells. Upon ligand binding, PDGF-BB induces receptor dimerization and autophosphorylation, which subsequently triggers downstream intracellular signaling cascades to precisely regulate core physiological processes such as cell proliferation, differentiation, and migration (Barrientos et al., 2008; Wang et al., 2019; Guerit et al., 2021).

Aging is a progressive biological process driven by the time-dependent accumulation of molecular and cellular damage, characterized by homeostatic imbalance, organ system decline, compromised stress resilience, and an exponential increase in susceptibility to chronic diseases (Lopez-Otin et al., 2023). Emerging evidence positions PDGF-BB as a pivotal mediator linking senescent phenotypes to various pathological conditions (Liu et al., 2023; Santhanam et al., 2021; Zhang et al., 2025). However, the expression profiles of PDGF-BB exhibit substantial heterogeneity within aging tissues and distinct disease microenvironments. Although the precise *in vivo* concentration threshold of PDGF-BB remains incompletely defined, its biological functions are profoundly dose-dependent. Evidence demonstrates that PDGF-BB operates at distinct magnitudes under physiological versus pathological conditions, frequently exerting diametrically opposing effects on identical cell types (Santhanam et al., 2021; Xie et al., 2014; Chen et al., 2015). While moderate levels generally support tissue homeostasis and anabolism (Xie et al., 2014; Chen et al., 2015), pathological hyper-accumulation abruptly shifts its role to drive degenerative processes such as bone loss and vascular remodeling (Santhanam et al., 2021; Zaidi et al., 2021). This biphasic nature adds a critical layer of complexity to its biological regulation. The precise molecular mechanisms underlying its mediation of pathological damage remain partially understood, and clinical strategies targeting PDGF-BB warrant further robust evidence-based validation. To address these gaps, this review systematically focuses on three core dimensions: (1) the distinct expression patterns of PDGF-BB in aging-related diseases and other pathological states; (2) the underlying molecular mechanisms dictating its biological functions; and (3) the development and translational potential of therapeutics targeting PDGF-BB pathways. Ultimately, this comprehensive overview aims to provide a theoretical foundation for optimizing the diagnosis and treatment of these disorders. To achieve this objective, a literature search of the PubMed database was conducted. The search strategy utilized the keyword combinations (PDGF-BB AND aging) OR (PDGF-BB AND senescence). Initial screening was performed by assessing titles and abstracts, followed by a full-text review. Studies were excluded if they did not directly investigate the expression profiles, pathophysiological mechanisms, or therapeutic potential of PDGF-BB in the context of aging and age-related pathologies.

## 2 Expression profiles of PDGF-BB in aging-related diseases and pathological states

Alterations in PDGF-BB expression exhibit pronounced tissue specificity and disease dependency. During the natural aging process, its aberrant expression often acts as a critical driver contributing to the pathophysiology of specific age-related diseases. Conversely, existing pathological conditions can reciprocally disrupt PDGF-BB homeostasis. This bidirectional relationship underscores the central regulatory role of PDGF-BB in both maintaining physiological stability and driving pathological progression, a dynamic orchestrated by the tissue-specific activation of regulatory pathways, alterations in aging-associated hormonal microenvironments, and the specific nature of pathological stimuli. To facilitate cross-comparison and enhance clarity, the heterogeneous expression patterns of PDGF-BB across distinct tissues, aging contexts, and disease states are systematically summarized in Table 1.

**TABLE 1:** Spatiotemporal expression profiles of PDGF-BB across aging and pathological states.

Aging phenotype/Disease context	Sample source	Expression pattern	Corresponding references
Systemic Aging Dynamics	Serum	Controversial (Decreased after early adulthood vs. Elevated in elderly)	(Santhanam et al., 2021; Evanson et al., 2014; Tang et al., 2017)
Arterial Aging and Stiffening	Aortic tissue	Elevated	(McCaffrey et al., 1988; Wang et al., 2018)
Vascular Calcification (HFD-induced)	Serum	Elevated	(Santhanam et al., 2021)
Pulmonary Arterial Hypertension (PAH)	PASMCs/Lung circulation	Elevated	(Selimovic et al., 2009; Perros et al., 2008)
Atherosclerotic Cerebrovascular Disease (CeVD)	Plasma (Endothelial cell-derived exosomes)	Elevated	(Goetzl et al., 2017)
Hippocampal Vasculature and BBB Dysfunction	Serum (Bone-derived)	Elevated	(Liu et al., 2023)
Alzheimer’s Disease (AD) Pathology	Brain tissue	Elevated	(Masliah et al., 1995)
Alzheimer’s Disease (AD) Resilience	Brain tissue	Elevated	(Barroeta-Espar et al., 2019)
Aging-induced Osteoporosis	Serum (Preosteoclast-derived)	Elevated	(Santhanam et al., 2021; Zaidi et al., 2021)
Intervertebral Disc Degeneration (IDD)	Nucleus pulposus (NP)/Intervertebral disks (IVDs)	Elevated	(Zhang et al., 2021; Zheng et al., 2024)
Non-alcoholic Steatohepatitis (NASH)	Hepatic tissue	Elevated	(Aljabban et al., 2022)
Liver Fibrosis (BDL-induced)	Serum	Elevated	(Ogawa et al., 2010)
NAFLD/HCC (with *PNPLA3* Mutation)	Serum	Decreased	(Kirchmeyer et al., 2023)
Lymph Node-Metastatic Cholangiocarcinoma (CCA)	Cancer-associated fibroblasts (CAFs)	Elevated	(Yan et al., 2024)
Esophageal Squamous Cell Carcinoma (ESCC)	Serum	Elevated	(Er et al., 2020)
Diminished Ovarian Reserve (DOR)	Follicular fluid	Decreased	(Bouet et al., 2020)
Sarcopenia and Physical Frailty	Serum	Decreased	(Marzetti et al., 2019; Calvani et al., 2021; Picca et al., 2020)

Before delving into specific disease contexts, it is crucial to explicitly distinguish between local tissue-specific PDGF-BB expression and circulating systemic PDGF-BB levels, as their biological implications are fundamentally distinct. Local PDGF-BB expression primarily reflects highly localized autocrine and paracrine signaling cascades orchestrated by specific cell types to maintain niche homeostasis or drive focal pathological remodeling. In contrast, circulating PDGF-BB concentrations measured in serum or plasma represent a systemic integration of diverse biological processes. These systemic levels may indicate generalized platelet activation, overall inflammatory status, or the substantial endocrine spillover from a specific hyperactive local niche, such as the osteoporotic bone marrow. Therefore, interpreting disease mechanisms requires a strict conceptual separation of these two pools. Local expression drives site-specific cellular phenotypes, whereas alterations in circulating levels govern systemic cross-organ communication and often serve as accessible clinical biomarkers.

### 2.1 Serum PDGF-BB dynamics during natural aging

The precise trajectory of serum PDGF-BB levels in naturally aging populations remains controversial. Certain population-based studies indicate that circulating PDGF-BB concentrations decrease after early adulthood, specifically around 25 years of age (Evanson et al., 2014). However, evidence supporting a continuous progressive decline throughout middle and advanced age remains insufficient. Interestingly, this factor exhibits a positive correlation with serum estradiol concentrations in women (Tang et al., 2017). Conversely, distinct cohort analyses have documented elevated serum PDGF-BB levels in elderly individuals (Santhanam et al., 2021). These discrepancies strongly indicate that the heterogeneity in current findings stems from profound variations in baseline characteristics across different study populations. Specifically, inconsistencies in defined age ranges and sex distributions fundamentally alter the physiological baseline, as evidenced by the strict dependence of PDGF-BB on estradiol levels in postmenopausal women. Furthermore, the presence of subclinical comorbidities such as early-stage vascular stiffness or metabolic syndrome, concurrent medication use including antihypertensive or lipid-lowering therapies, and diverse lifestyle factors such as dietary habits and physical activity introduce significant confounding variables. When left uncontrolled, these multifaceted factors interact synergistically to obscure the true physiological trajectory of systemic PDGF-BB during natural aging. To provide a clearer comparative perspective, Table 2 systematically summarizes the key studies investigating serum PDGF-BB dynamics, highlighting the methodological and demographic variances that may account for these conflicting results. As clearly demonstrated by the drastic differences in sample sizes, sex ratios, and clinical inclusion criteria across the studies in the table, demographic and clinical heterogeneities are the primary drivers of the observed discrepancies. Furthermore, while transcriptomic databases, such as CircaDB, reveal diurnal oscillations in *Pdgfb* expression across diverse mouse tissues (Pizarro et al., 2013), these preclinical transcript-level variations cannot be directly extrapolated to significant diurnal fluctuations in human circulating PDGF-BB protein levels. Therefore, although theoretical circadian dynamics might represent an additional biological variable, they are largely overshadowed by the profound cohort heterogeneities described above.

**TABLE 2:** Summary of key studies investigating serum PDGF-BB levels in aging populations.

References	Total participants	Age groups	Clinical characteristics	Reported PDGF-BB Trend
(Santhanam et al., 2021)	15 (6 males, 9 females)	Young 21–26 years (3 females 2 males) Old 58–71 years (6 females 4 males)	Strictly healthy excluding obesity diabetes hypertension and vascular disorders	Elevated in elderly individuals
(Evanson et al., 2014)	102 (71 males, 31 females)	17–60 years old analyzed <25 (N = 62) and >25 (N = 38) subgroups	Healthy blood donors	Significantly higher in individuals <25 years old
(Tang et al., 2017)	121 females	Young women 20–48 years and postmenopausal women 45–76 years	Healthy young women postmenopausal controls and postmenopausal osteoporosis patients	Inversely correlated with age and positively correlated with estradiol with the lowest levels in osteoporosis patients

### 2.2 Vascular aging and pathological remodeling

Aging induces intimal thickening of the arterial wall, a senescent process intimately linked to the activation of VSMCs and further accelerated by PDGF signaling via the promotion of VSMC proliferation and migration (Yang et al., 2009; Raines et al., 2000; Ha et al., 2015; Wang et al., 2015). Studies have demonstrated that the aberrant proliferation of aortic smooth muscle cells in aged animals correlates directly with elevated local PDGF expression (McCaffrey et al., 1988). Specifically, PDGF-BB is markedly upregulated in the aortic wall of aged rats (Wang et al., 2018). However, caloric restriction interventions can effectively suppress this upregulation, thereby attenuating VSMC migration and subsequent fibrosis (Wang et al., 2018). Furthermore, systemic PDGF-BB levels remain persistently elevated in both aged mice and high-fat diet (HFD)-induced disease models (Santhanam et al., 2021). Notably, an upregulation of preosteoclast-derived PDGF-BB can trigger aortic lumen dilation, wall thickening, and vascular calcification (Santhanam et al., 2021). It is crucial to explicitly highlight the discrepancies regarding the primary cellular sources of PDGF-BB driving these vascular phenotypes across different studies. For instance, while research by Wang and colleagues demonstrates that PDGF-BB is locally produced within the arterial wall to drive local tissue remodeling (Wang et al., 2018), Santhanam and colleagues provide compelling evidence that circulating PDGF-BB is primarily derived from preosteoclasts in the bone and bone marrow (Santhanam et al., 2021). Rather than being mutually exclusive, these contradictory findings suggest that vascular stiffening is orchestrated by a dual mechanism. Localized arterial wall expression likely governs autocrine and paracrine maintenance, whereas bone-derived systemic spillover mediates endocrine cross organ pathological communication. Acknowledging such source heterogeneity is essential for fully comprehending the diverse and highly contextual roles of PDGF-BB.

In a parallel context, circulating PDGF-BB levels are significantly elevated in patients with pulmonary arterial hypertension (PAH) (Selimovic et al., 2009). Within the pulmonary arterial smooth muscle cells (PASMCs) of these patients, the expression of PDGF-A, PDGF-B, and their cognate receptors is significantly upregulated (Perros et al., 2008). This implies that pathological stimuli may provoke the autonomous secretion of PDGF-BB by PASMCs, establishing an autocrine loop that drives aberrant cell proliferation and phenotypic switching, which ultimately exacerbates pulmonary vascular remodeling (Rabinovitch, 2012). Similarly, in patients with atherosclerotic cerebrovascular disease (CeVD), the PDGF-BB payload within endothelial cell-derived exosomes is substantially enriched, further demonstrating the widespread involvement of this altered growth factor in localized vascular pathogenesis (Goetzl et al., 2017).

Collectively, these findings underscore that the dysregulation of PDGF-BB expression actively participates in the pathological progression of cardiovascular and cerebrovascular conditions, including arterial stiffness, pulmonary hypertension, and atherosclerosis, thereby establishing its role as an important mediator of vascular aging.

### 2.3 Neurovascular unit dysfunction and neurodegeneration

Within the spectrum of age-related cognitive decline and neurodegenerative disorders such as Alzheimer’s disease (AD), the heterogeneous expression of PDGF-BB underscores the intricate nature of the underlying pathologies (Liu et al., 2023; Masliah et al., 1995). While VSMCs orchestrate pathological remodeling in large arteries, capillary pericytes serve a pivotal and distinct function within the microcirculation. Anatomically positioned at the frontline of blood-tissue exchange, pericytes intimately surround microvascular endothelial cells to govern blood-brain barrier (BBB) integrity and local hemodynamics (Liu et al., 2023). Crucially, this unique spatial localization renders them highly susceptible to systemic fluctuations in circulating PDGF-BB ligands. During the aging process, aberrant elevations in serum PDGF-BB, which often originate from distant pathological niches such as the osteoporotic skeleton, act as a chronic pathogenic mediator (Santhanam et al., 2021; Zaidi et al., 2021). This chronic ligand exposure directly impinges upon the vulnerable microvascular niche. In aged mouse models, such heightened systemic exposure has been shown to disrupt pericyte survival signaling, inducing substantial pericyte loss and diminishing capillary density within the hippocampus (Liu et al., 2023). This cascade consequently compromises BBB integrity and perturbs the homeostasis of the neural microenvironment, firmly establishing pericyte vulnerability as a critical node linking systemic circulatory changes to neurodegeneration (Liu et al., 2023).

In contrast, analyses of human brain tissues exhibiting AD pathological resilience reveal a distinct cytokine expression profile. These resilient individuals harbor a high neuropathological burden yet remain cognitively intact, suggesting that specific inflammatory mediators may confer neuroprotection. Compared to typical symptomatic AD patients, these resilient cohorts demonstrate a significant downregulation of pro-inflammatory chemokines (e.g., MCP-1 and MIP-1α), juxtaposed with a robust upregulation of growth factors equipped with neurotrophic and regenerative properties, notably including PDGF-BB and basic fibroblast growth factor (bFGF) (Barroeta-Espar et al., 2019). This stark dichotomy strongly implies that PDGF-BB can exert diametrically opposed effects contingent upon the specific neuropathological microenvironment it occupies.

### 2.4 Skeletal aging and degenerative bone/disc diseases

PDGF-BB exhibits a paradigmatic dual-regulatory profile in bone metabolism, characterized by strict dose dependency and source specificity. During aging-induced osteoporosis, the aberrant secretion of PDGF-BB by preosteoclasts dampens the osteogenic capacity of osteoblasts and elevates systemic PDGF-BB levels, thereby accelerating bone loss (Zaidi et al., 2021). In contrast, in young organisms, PDGF-BB typically exerts positive anabolic effects on bone metabolism. In fracture models, for instance, Omentin-1 mitigates inflammation and stimulates PDGF-BB secretion from preosteoclasts, inducing the formation of CD31hiEmcnhi (type H) vessels to synergistically couple angiogenesis with osteogenesis for optimal fracture healing (Feng et al., 2021). Furthermore, in ovariectomized (OVX) osteoporotic models, myeloid-specific deletion of *Msx2* induces robust PDGF-BB expression in osteoclast precursors, consequently promoting type H vessel formation and bone mass accretion (Ma et al., 2025). These findings indicate that age-related disparities in the bone marrow microenvironment fundamentally influence the divergent regulatory effects of PDGF-BB.

Precise manipulation of *in vivo* PDGF-BB concentrations using conditional gene editing technologies reveals an exceptionally narrow physiological therapeutic window for maintaining skeletal integrity. Within this window, PDGF-BB acts as the central mediator coupling angiogenesis with osteogenesis. However, once this threshold is breached or its cellular source is misplaced, it transforms into an accelerator for bone loss. Evidence demonstrates that osteoclast-specific knockout of *Pdgfb* precipitates a severe decline in systemic levels and induces osteoporosis (Xie et al., 2014). Conversely, driving its pathological accumulation via specific overexpression equally triggers the collapse of bone homeostasis (Santhanam et al., 2021). In stark contrast to these extreme phenotypes, employing a weaker PGK promoter—rather than a robust SFFV promoter—to drive merely moderate *Pdgfb* overexpression in bone marrow mesenchymal stem cells (BMSCs) effectively promotes osteogenic differentiation and augments bone mineral density (Chen et al., 2015). This profound dose-dependency highlights that preserving the physiological concentration gradient of PDGF-BB is paramount for skeletal integrity. Consequently, this implies that in the clinical management of the aging skeleton, crude systemic supplementation or blockade is unlikely to be effective; instead, success hinges on highly targeted, spatially specific delivery strategies.

During the pathological progression of intervertebral disc degeneration (IDD), PDGF-BB is highly expressed within the local microenvironment of the nucleus pulposus (NP) (Zhang et al., 2021). Cells within the degenerated tissue upregulate PDGF-BB to activate downstream MAPK signaling, which serves to inhibit NP cell pyroptosis and thereby retards the degenerative process (Zhang et al., 2021). Supplementation with exogenous recombinant PDGF-BB downregulates the expression of senescence-associated genes in NP cells, unveiling its latent anti-aging therapeutic potential within the intervertebral disc (IVD) (Zhang et al., 2025). A deficiency in macrophage-derived PDGF-BB exacerbates the loss of NP cells and heightens cartilage endplate ossification during lumbar disc degeneration, ultimately accelerating IDD progression (Zheng et al., 2024). These collective observations suggest that the upregulation of PDGF-BB under IVD pathological conditions likely represents a compensatory mechanism orchestrated to promote tissue repair.

### 2.5 Hepatic aging and associated liver diseases

During hepatic aging and the pathogenic progression from non-alcoholic fatty liver disease (NAFLD) to non-alcoholic steatohepatitis (NASH) and subsequent liver fibrosis, PDGF-BB plays a pivotal pro-fibrotic role (Borkham-Kamphorst and Weiskirchen, 2016; Tsuchida and Friedman, 2017; Czochra et al., 2006). Clinical transcriptomic analyses have confirmed a marked upregulation of localized hepatic PDGF-BB expression in patients with NASH, a pathological event that coincides with the robust activation of SREBF1-driven lipid metabolic networks during the NAFLD stage (Aljabban et al., 2022).

Nevertheless, PDGF-BB levels within the systemic circulation exhibit profound complexity. While serum PDGF-BB concentrations rise substantially in bile duct ligation (BDL)-induced murine models of liver fibrosis (Ogawa et al., 2010), peripheral PDGF-BB levels undergo a notable decline in NAFLD or hepatocellular carcinoma (HCC) patients harboring the *PNPLA3* genetic mutation (Kirchmeyer et al., 2023). The juxtaposition of elevated localized tissue transcription and diminished peripheral serum levels under specific genotypic backgrounds underscores that the expression and regulation of this specific PDGF isoform during hepatic pathogenesis are stringently and intricately orchestrated by both lipid metabolic status (such as the *SREBF1* pathway) and genetic polymorphisms (such as the *PNPLA3* mutation). Furthermore, potential confounding inputs from PDGF-BB secreted by extrahepatic tissues into the systemic pool cannot be ruled out. Consequently, when evaluating PDGF-BB as a therapeutic target or clinical biomarker, it is imperative to rigorously account for the spatial compartmentalization between the hepatic microenvironment and the peripheral circulation, while fully integrating the patient’s genetic background.

### 2.6 Tumor microenvironment and malignant progression

Aging-associated microenvironmental dysregulation serves as a potent catalyst for oncogenesis. In patients with lymph node-metastatic cholangiocarcinoma (CCA), cancer-associated fibroblast (CAF)-derived PDGF-BB robustly accelerates lymphangiogenesis and tumor cell migration (Yan et al., 2024). Similarly, serum PDGF-BB levels are markedly elevated in individuals with locally advanced esophageal squamous cell carcinoma (ESCC), a phenotype that not only correlates with an unfavorable prognosis but also directly enhances tumor cell proliferation and radioresistance (Er et al., 2020).

### 2.7 Reproductive senescence and musculoskeletal degeneration

Aberrant PDGF-BB expression similarly acts as a critical determinant in reproductive and musculoskeletal aging. In women with diminished ovarian reserve (DOR), PDGF-BB levels within the follicular fluid undergo a substantial decline, suggesting that a localized deficiency of this growth factor may contribute to the pathogenesis of ovarian senescence (Bouet et al., 2020). Correspondingly, circulating PDGF-BB concentrations in elderly patients with sarcopenia are significantly lower than those in age-matched healthy peers, implying a tight linkage between systemic PDGF-BB deficiency and age-related muscle mass loss or functional decline (Marzetti et al., 2019). Recently, multi-marker approaches utilizing multicenter clinical cohorts, such as the BIOSPHERE study, have unveiled the unique systemic hallmarks of physical frailty and sarcopenia (PF&S). These investigations demonstrate that, distinct from mere chronic inflammatory accumulation, the pathogenesis of PF&S hinges on the collapse of tissue regenerative capacity and stress adaptation. For healthy elderly populations, maintaining elevated circulating levels of PDGF-BB (which provides regenerative and vasculotrophic signals) alongside heat shock protein 72 (HSP72, which sustains systemic antioxidant defense) constitutes a pivotal protective biomarker network that safeguards against the development of PF&S (Calvani et al., 2021; Picca et al., 2020).

## 3 Mechanistic roles of PDGF-BB in aging and pathological remodeling

The biological execution of PDGF-BB relies on an intricate cascade of events encompassing receptor engagement, signal transduction, phenotypic modulation, and intercellular communication. This mechanistic framework exhibits pronounced source specificity, dose dependency, and target-cell selectivity. Systemically, PDGF-BB acts as a hub for inter-organ crosstalk. Intracellularly, it drives kinase-mediated aberrant cellular proliferation and phenotypic switching, inflammation, autophagic metabolic switching, and pathological extracellular matrix (ECM) remodeling. Concurrently, it regulates malignant microenvironments and preserves stem cell homeostasis. Fine-tuned by upstream epigenetic circuits, PDGF-BB ultimately serves as a central mediator of aging-induced degenerative disorders.

### 3.1 Intercellular communication and inter-organ crosstalk

Depending on the specific microenvironmental context, PDGF-BB can exert pronounced protective and reparative effects. In the context of cell senescence regulation, for instance, locally secreted PDGF-BB from BMSCs effectively shields target cells from apoptosis and senescence by directly suppressing the canonical p53/p21 senescence signaling axis (Zhang et al., 2016). Similarly, during physiological fracture healing, PDGF-BB derived from pre-osteoclasts induces the formation of localized CD31hiEmcnhi (type H) vessels, thereby providing essential vascular network support and a pro-osteogenic microenvironment for the injured region (Feng et al., 2021).

Under conditions of tissue aging, PDGF-BB mediated intercellular communication emerges as a critical nexus linking local microenvironmental decompensation to systemic disease. As previously detailed regarding the bone-vascular axis, the systemic spillover of preosteoclast-derived PDGF-BB directly orchestrates a seamless inter-organ pathological coupling between localized skeletal aging and systemic arterial stiffening (Zaidi et al., 2021; Su et al., 2025). Recent studies further reveal that the systemic microenvironment in osteoporotic individuals undergoes profound remodeling, characterized by altered circulating immune cell profiles alongside an aberrant enrichment of factors such as PDGF-BB and osteopontin (Pustlauk et al., 2025). *In vitro* experiments have corroborated that this complex osteoporotic milieu acts as an independent risk factor for vascular calcification, directly accelerating the pathological calcification of human vascular smooth muscle cells. Notably, targeted blockade of PDGF-BB downstream signaling effectively reverses this effect (Pustlauk et al., 2025). This discovery inextricably links osteoporosis-induced bone loss to systemic arterial stiffness, firmly establishing PDGF-BB as a critical link in the cross-organ pathological communication of the bone-vascular axis.

Beyond driving vascular degeneration, the systemic accumulation of bone-derived PDGF-BB triggers pathological bone-brain crosstalk, thereby impairing neurovascular homeostasis. Evidence confirms that excessive serum PDGF-BB upregulates MMP14 expression in brain microvascular pericytes, triggering the shedding of surface PDGFRβ (Liu et al., 2023). This directly compromises BBB integrity and damages the hippocampal capillary network, tightly coupling aberrant bone metabolism with neurocognitive decline. Concurrently, bone-derived PDGF-BB infiltrating the central nervous system provokes the nuclear translocation of specificity protein 1 (SP1) within endothelial cells, drastically exacerbating transcytosis and neuroinflammation in the hippocampus (Liu et al., 2024), and promoting cerebrovascular calcification via the activation of the ERK pathway (Wang et al., 2023), thereby accelerating the age-related degeneration of brain tissues.

### 3.2 Intracellular signaling networks and cellular phenotypic switching

PDGF-BB stands as the sole ligand capable of binding and activating all three receptor dimeric configurations (PDGFR-αα, PDGFR-αβ, and PDGFR-ββ) with high affinity. This unique biochemical feature serves as the structural foundation for the profound heterogeneity and pleiotropy observed in its downstream biological effects (Fredriksson et al., 2004; Andrae et al., 2008). It is imperative to distinguish the specific actions of this unique ligand from general receptor activation. While the subsequent intracellular cascades, including ERK, p38, JNK, mTOR, PI3K-AKT, and NF-κB, represent broad signaling networks shared by numerous unrelated growth factor families, the spatiotemporal specificity of PDGF-BB is largely governed by its universal receptor engagement pattern. Notably, during the aging process, the hyperactivation of these specific downstream cascades by PDGF-BB closely intertwines with the established hallmarks of aging, directly driving tissue degeneration.

Before these intracellular cascades engage, PDGF-BB signaling is dynamically gated at the receptor level. Beyond classical membrane-bound dimers, PDGFR genetic mutations can fundamentally alter baseline cellular responsiveness and drive hyper-proliferative disorders (Guerit et al., 2021). Furthermore, soluble receptor isoforms, particularly soluble PDGFR (sPDGFRβ), exhibit a highly contextual duality. Pathologically, vascular pericytes shed membrane-bound PDGFRβ, releasing sPDGFRβ into the cerebrospinal fluid (CSF) (Sagare et al., 2015). Consequently, elevated CSF sPDGFRβ serves as a primary biomarker for BBB breakdown, correlating directly with decreased resting-state functional connectivity in the default mode network (DMN) of older adults (Contreras et al., 2024). However, sPDGFRβ is not merely a passive injury byproduct. Physiologically, it is endogenously produced via pre-mRNA alternative splicing (Payne et al., 2023). These native isoforms act as decoy receptors to sequester extracellular PDGF-BB, creating a physiological buffer that prevents signaling hyperactivation and maintains microenvironmental homeostasis, a balance frequently disrupted during aging and hypoxia (Payne et al., 2023). Recognizing this dual identity of PDGFRβ variants as both damage biomarkers and homeostatic modulators clarifies how PDGF-BB signal intensity is calibrated. Once this receptor-level gating is bypassed or overwhelmed, PDGF-BB vigorously drives downstream intracellular networks.

In the context of vascular aging, a central mechanism by which PDGF-BB drives pathological remodeling is through the induction of aberrant cellular phenotypic switching (Ha et al., 2015), which directly fuels the amplification of vascular inflammaging (Yang et al., 2009; Wang et al., 2014). Experimental studies indicate that PDGF-BB-driven VSMC proliferation and migration are heavily dependent on the ERK, p38, and JNK signaling pathways (Zhan et al., 2003). Crucially, the sustained activation of these cascades, particularly the ERK and p38 axes, forces VSMCs to adopt a highly secretory synthetic phenotype. During the aging process, this synthetic state closely mirrors the senescence-associated secretory phenotype (SASP), directly contributing to vascular inflammaging and the localized accumulation of pro-inflammatory cytokines (Wang et al., 2014). Furthermore, targeted blockade of the mTOR/Drp1 pathway effectively suppresses PDGF-BB induced smooth muscle cell hyperplasia (Chen et al., 2020), highlighting the PDGF-BB/mTOR axis as a pivotal pathway in driving age-related vascular stiffness rather than merely a general proliferative mechanism.

Driven by the synergistic disruption of kinase signaling and autophagic metabolic homeostasis, the progressive decline in parenchymal regenerative capacity frequently shifts tissue responses from physiological healing to pathological fibrosis, representing a classic manifestation of impaired tissue repair (Kisseleva and Brenner, 2021). Within this deteriorated age-related microenvironment, PDGF is recognized as the most potent mitogen priming hepatic stellate cells (HSCs) for hyperactivation (Pinzani et al., 1989). Upon stimulation, PDGF-BB robustly drives their aberrant proliferation and directed migration via the classical ERK pathway (Rovida et al., 2008) and the FAK/PI3K/AKT signaling axis (Reif et al., 2003). Crucially, to satisfy the heightened energetic demands required for this pathological phenotypic switching, PDGF-BB aberrantly manipulates the endogenous autophagic machinery within HSCs. Driven by PDGF-BB signaling, specifically through the dynamic modulation of the mTOR pathway, the induction of autophagic activation not only sustains their hyperactive state but also provokes the robust paracrine release of miR-29a-containing vesicles, further accelerating the pathological progression of liver fibrosis (Yu et al., 2022). Intriguingly, this autophagic flux possesses a highly dynamic, double-edged regulatory threshold. Evidence indicates that under specific interventional contexts, such as the targeted silencing of the endoplasmic reticulum protein Nogo-B, pushing the autophagic flux beyond its adaptive limit can forcibly expedite the senescence of PDGF-BB-induced HSCs, effectively halting their pathological proliferation and blunting fibrotic progression (Gao et al., 2025). Through the synergistic orchestration of kinase cascades and profound autophagic metabolic reprogramming, PDGF-BB comprehensively drives the pathological activation of HSCs, ultimately exacerbating age-related hepatic microenvironmental deterioration.

This aberrant activation of the ERK signaling pathway also serves as a central mechanism driving pathological ECM remodeling, a core feature of organ aging. The excessive accumulation and cross-linking of ECM proteins severely compromise tissue compliance and accelerate functional decline across diverse organs (Fane and Weeraratna, 2020). In age-related renal diseases, for instance, PDGF-BB has been similarly proven to drive DNA synthesis and ECM remodeling in mesangial cells by activating the ERK signaling axis (Kagami et al., 2004). Furthermore, during the progression of arterial aging, PDGF-BB signaling is a potent driver of vascular wall stiffening. Beyond promoting cellular phenotypic switching, the PDGF-BB/ERK cascade directly stimulates the robust synthesis and deposition of profibrotic ECM components, such as collagen type I and fibronectin, profoundly accelerating the age-related fibrotic remodeling of the vascular matrix (Wang et al., 2015).

Crucially, the robust orchestrating capabilities of PDGF-BB are frequently hijacked within the complex tumor microenvironment, a process that is profoundly exacerbated by inflammaging and immune senescence. During aging, the persistent systemic accumulation of diverse senescent cell populations produces a chronic, low-grade inflammatory milieu, characterized by the robust senescence-associated secretory phenotype (SASP). This systemic inflammaging severely compromises host tissue surveillance and establishes a broadly immunosuppressive, tumor-permissive niche (Franceschi et al., 2018; Campisi, 2013). Within this deteriorated age-related microenvironment, host immune cells are frequently reprogrammed to fuel disease progression. For instance, in malignancies such as colorectal cancer (CRC), macrophages are polarized into M2-type tumor-associated macrophages (TAMs), becoming a primary source of abundant paracrine PDGF-BB secretion (Guo et al., 2024). Driven by this localized hyperaccumulation, PDGF-BB robustly activates dual intracellular signaling axes to drive malignant cellular reprogramming. On one hand, it activates ERK1/2-JNK signaling in surrounding lymphatic endothelial cells to stimulate chaotic angiogenesis and lymphangiogenesis. Concurrently, it triggers a parallel GSK-3β/p65 axis directly within tumor cells to upregulate their invasive and proliferative potential (Yan et al., 2024). Together, these mechanisms demonstrate how the hyperactivation of specific intracellular kinase networks by reprogrammed immune cells acts as a critical molecular transducer, effectively linking age-associated chronic inflammation to malignant microenvironmental remodeling.

Parallel to these mechanisms, the PI3K-AKT pathways occupy a dominant role in dictating cellular survival and combating stem cell dysfunction during natural aging. Research indicates that BMSC-derived PDGF-BB can significantly enhance the viability of progenitor cells by activating the downstream PI3K-AKT-MEK/ERK signaling cascades thus preventing premature cellular senescence and preserving tissue regenerative capacity (Fang et al., 2020).

### 3.3 Epigenetic and post-transcriptional regulation

Amidst the complexities of age-related microenvironmental changes, epigenetic modifications and post-transcriptional regulatory networks function as a critical upstream fine-tuning system that dictates the magnitude of PDGF-BB signaling. During the aging process, the progressive decline of endogenous signaling repressors, specifically microRNAs, leads to the unconstrained hyperactivation of PDGF-BB and its downstream effectors, profoundly accelerating tissue degeneration. During vascular remodeling, for instance, miR-223 specifically targets the transcription factor *NFAT5*, thereby potently abrogating the aberrant proliferation of smooth muscle cells induced by localized PDGF-BB (Su et al., 2020). In a parallel manner within pulmonary arterial hypertension (PAH) models, miR-30 d obstructs the MTDH/PDE5A signaling axis, significantly attenuating PDGF-BB-mediated hyperplasia in pulmonary arterial smooth muscle cells (Liang et al., 2024). The dysregulation of these endogenous post-transcriptional regulatory networks likely represents a critical predisposing factor for the hyperactivation of PDGF-BB signaling cascades and subsequent tissue degeneration.

Crucially, PDGF-BB-mediated phenotypic reprogramming relies not only on the immediate propagation of intracellular kinase cascades but also deeply penetrates the epigenetic landscape, thereby hardwiring the pathological traits of target cells at the transcriptional level. Evidence indicates that PDGF-BB stimulation robustly induces the upregulation and aberrant activation of histone deacetylase 4 (HDAC4). As a pivotal epigenetic modifier, the pathological enrichment of HDAC4 directly drives the dedifferentiation, hyperproliferation, and directed migration of VSMCs, cementing its role as a core epigenetic target in vascular lesions such as arterial neointimal hyperplasia (Usui et al., 2014). Notably, these PDGF-BB-driven epigenetic modifications are subjected to rigorous surveillance by upstream non-coding RNA networks. The introduction of specific miRNAs, such as miR-149–5p, to silence HDAC4 expression can effectively arrest PDGF-BB-induced VSMC invasion and malignant proliferation (Zhang et al., 2019). These findings indicate that the profound coupling among kinase signaling, non-coding RNAs, and epigenetic modifications constitutes the comprehensive molecular network through which PDGF-BB drives disease progression.

## 4 Therapeutic interventions targeting PDGF-BB and their translational implications

Driven by the pivotal regulatory function of PDGF-BB in aging and pathogenesis, therapeutic strategies designed to modulate its expression levels or intercept its signaling cascades have emerged as a prominent research Frontier. The current pharmacological arsenal primarily encompasses small-molecule compounds, natural products, and biological agents.

### 4.1 Small-molecule compounds and natural products

Current small-molecule interventions targeting PDGF-BB generally follow two distinct therapeutic trajectories: upregulating endogenous expression to promote tissue repair, or intercepting downstream signaling to halt pathological proliferation. In terms of promoting osteovascular repair, natural products exhibit a unique advantage by remodeling the microenvironment. For example, both morusin and harmine can intervene in the transcriptional network of preosteoclasts, effectively restoring the physiological secretion of PDGF-BB in aged bone tissue to ameliorate osteoporosis (Ma et al., 2025; Huang et al., 2021). Conversely, when addressing vascular proliferative diseases, the current interventional focus often relies on uncoupling downstream kinase cascades. Whether utilizing canagliflozin to target the SGLT1/AMPK axis or corylin to obstruct the mTOR/Drp1 pathway, the rationale lies in suppressing hyperactivated signaling to deprive smooth muscle cells of their pathological proliferative drive (Chen et al., 2020; Chen et al., 2024). However, it must be explicitly noted that these interventions inhibit broadly shared downstream kinase networks rather than targeting PDGF-BB specifically. Consequently, such broad-spectrum inhibition carries a significant risk of interfering with unrelated growth factor pathways. To establish PDGF-BB as a precise therapeutic target, future drug development must shift away from indiscriminate downstream kinase blockade and focus primarily on isolating highly specific ligand receptor interactions.

### 4.2 Biological agents and cell therapies

Targeting the PDGF-BB/PDGFR signaling axis has emerged as a pivotal therapeutic paradigm for developing anti-fibrotic agents and reversing disease progression. Studies indicate that mesenchymal stem cells (MSCs) preconditioned with IFN-γ and TNF-α express robust levels of PDGF-BB, demonstrating tangible translational value in accelerating chronic wound healing (Liu et al., 2022). Regarding targeted pharmacotherapy, neutralizing antibodies against PDGF-BB and PDGFR inhibitors (such as AG1296) have exhibited definitive interventional promise in abrogating tissue fibrosis and proliferative disorders (Ismaeel et al., 2022; Contreras et al., 2021). Furthermore, leveraging a weak phosphoglycerate kinase (PGK) promoter to drive the moderate, specific overexpression of *Pdgfb* in BMSCs effectively promotes their osteogenic differentiation and enhances bone mineral density (Chen et al., 2015).

Furthermore, recombinant PDGF-BB protein constitutes one of the most mature and promising directions for clinical translation. As extensively reviewed by Younan and colleagues, exogenous recombinant PDGF-BB has achieved significant clinical milestones, including FDA approvals for specific applications in tissue repair (Younan et al., 2026). Prominent examples include topical becaplermin for the treatment of diabetic lower extremity ulcers (Fang and Galiano, 2008) and recombinant human PDGF-BB combined with scaffolds for periodontal bone regeneration (Younan et al., 2026). These approved therapies leverage the potent mitogenic and angiogenic properties of PDGF-BB to accelerate localized wound healing and bone defect repair.

To circumvent the narrow therapeutic window and short biological half-life of PDGF-BB in osteovascular repair, intelligent biomaterial delivery systems integrated with tissue engineering represent the cutting edge of translational applications. For instance, a recently engineered trifunctional sialylated SF-ZIF@NA hydrogel system provides a potential targeted intervention strategy for osteoporotic bone defects. Upon enzymatic desialylation, this sophisticated material specifically blocks the fusion of preosteoclasts into mature multinucleated osteoclasts. This effectively suppresses aberrant bone resorption while maximally preserving the anabolic functions of the preosteoclasts. Consequently, these selectively retained preosteoclasts continuously and stably secrete high levels of endogenous PDGF-BB to activate the focal adhesion kinase (FAK) signaling pathway, ultimately achieving highly vascularized bone regeneration in osteoporotic bone defect models (Chen et al., 2025). This novel strategy of utilizing biomaterials for the *in situ* modulation of specific cell populations to dictate PDGF-BB release effectively circumvents the off-target effects and dose-limiting toxicities frequently associated with exogenous recombinant protein administration, thus exhibiting exceptional potential for clinical translation.

### 4.3 Translational applications and challenges

PDGF-BB harbors dual clinical utility, serving concurrently as a diagnostic biomarker and a therapeutic target. In the plasma of patients with atherosclerotic cerebrovascular disease (CeVD), the concentration of PDGF-BB within platelet-derived exosomes (PDEs) is significantly elevated, positioning it as a potential early diagnostic biomarker for CeVD (Goetzl et al., 2017). Furthermore, clinical investigations have revealed that in carriers of specific genotypes, elevated circulating PDGF-BB strongly correlates with white matter hyperintensity burden. This suggests that systemic PDGF-BB may serve as an indicative biomarker for blood-brain barrier disruption and the progression of vascular cognitive impairment (Kapoor and Nation, 2022). Clinical evidence also confirms that serum PDGF-BB concentration acts as an independent risk marker for predicting acute exacerbations and overall survival in patients with idiopathic pulmonary fibrosis (IPF) (Arai et al., 2022). Additionally, diminished serum PDGF-BB levels can be utilized as a metric to predict radiotherapy outcomes in ESCC patients (Er et al., 2020). Recent clinical trials evaluating the prognostic value of serum angiogenic factors in colorectal cancer explicitly indicate that, under specific phenotypic contexts, high concentrations of serum PDGF-BB correlate with more favorable prognoses, underscoring its utility as a prognostic stratification biomarker (Kasurinen et al., 2023). In postmenopausal osteoporosis, low plasma PDGF-BB levels are closely linked to estradiol concentrations (Tang et al., 2017), and its levels within the follicular fluid strongly correlate with ovarian reserve (Bouet et al., 2020). These findings collectively highlight PDGF-BB as a potent candidate biomarker for female aging.

To transition from preclinical models to real-world clinical applications, evaluating the outcomes of clinical trials targeting the PDGF-BB and PDGFR pathways is of paramount importance. Currently, broad-spectrum tyrosine kinase inhibitors with potent anti-PDGFR activity, such as imatinib and nintedanib, have generated substantial clinical data. In multicenter clinical trials for idiopathic pulmonary fibrosis and certain malignancies, these agents have demonstrated significant clinical efficacy in slowing disease progression and improving patient survival (Richeldi et al., 2014). However, these trials also vividly expose the inherent challenges of systemic PDGFR blockade. Because these small-molecule inhibitors frequently exhibit off-target affinity for other kinase receptors, patients commonly experience dose-limiting toxicities, including severe gastrointestinal adverse events, hepatotoxicity, and cardiovascular complications (Moslehi, 2016). Similarly, in clinical trials involving topical recombinant PDGF-BB therapy, such as becaplermin for diabetic lower extremity ulcers, achieving the optimal therapeutic dose remains a major clinical hurdle (Fang and Galiano, 2008). Epidemiological data from these trials indicate that excessive dosing or prolonged administration is linked to an elevated risk of localized hyperproliferation and secondary malignancies, forcing regulatory agencies to issue strict safety warnings (Fang and Galiano, 2008). Therefore, these clinical trial outcomes strongly emphasize that the future of PDGF-BB-targeted therapeutics depends critically on developing highly selective inhibitors or intelligent local delivery systems to uncouple therapeutic efficacy from systemic toxicity.

Despite these promising prospects, targeted therapies directed at PDGF-BB face several formidable challenges that urgently demand resolution: First, owing to its strict source and dose dependency, the therapeutic window is exceptionally narrow; both insufficient and excessive *in vivo* concentrations can inadvertently precipitate pathological states (Santhanam et al., 2021; Xie et al., 2014). Second, there is a current dearth of highly tissue- or cell-specific inhibitors, rendering systemic interventions highly susceptible to off-target effects. Third, existing clinical evidence remains relatively sparse, necessitating large-scale, multicenter clinical trials to rigorously validate both safety and efficacy. Fourth, the pleiotropic effects exerted by the same molecule derived from disparate cellular sources introduce profound biological complexities, thereby substantially escalating the difficulty of rational targeted drug design.

## 5 Discussion

As a quintessential pleiotropic growth factor, the biological functions of PDGF-BB in aging and pathogenesis are defined by pronounced source specificity, dose dependency, and contextual dependency. To address its fundamental role in aging biology, it is crucial to conceptually define PDGF-BB not as a static factor, but as a highly dynamic mediator whose function shifts along the disease continuum. In the context of acute tissue injury or early-stage localized degeneration, such as early intervertebral disc degeneration (Zhang et al., 2021; Zheng et al., 2024), it primarily emerges as a downstream compensatory repair signal aimed at restoring microenvironmental homeostasis. However, as chronological aging progresses and cellular damage accumulates, the sustained dysregulation and pathological hyperaccumulation of PDGF-BB abruptly transform it into an active upstream driver of systemic aging pathologies, as elegantly demonstrated by its role in driving cross-organ vascular stiffening (Santhanam et al., 2021; Zaidi et al., 2021). Concurrently, its spillover into the systemic circulation frequently allows it to act as a downstream biomarker reflecting cumulative tissue damage. Mechanistically, by orchestrating PDGFR-mediated signaling networks and integrating intricate inter-organ crosstalk mechanisms, PDGF-BB achieves precise spatiotemporal control over both systemic homeostasis and local microenvironmental stability (Figure 1).

**FIGURE 1:**
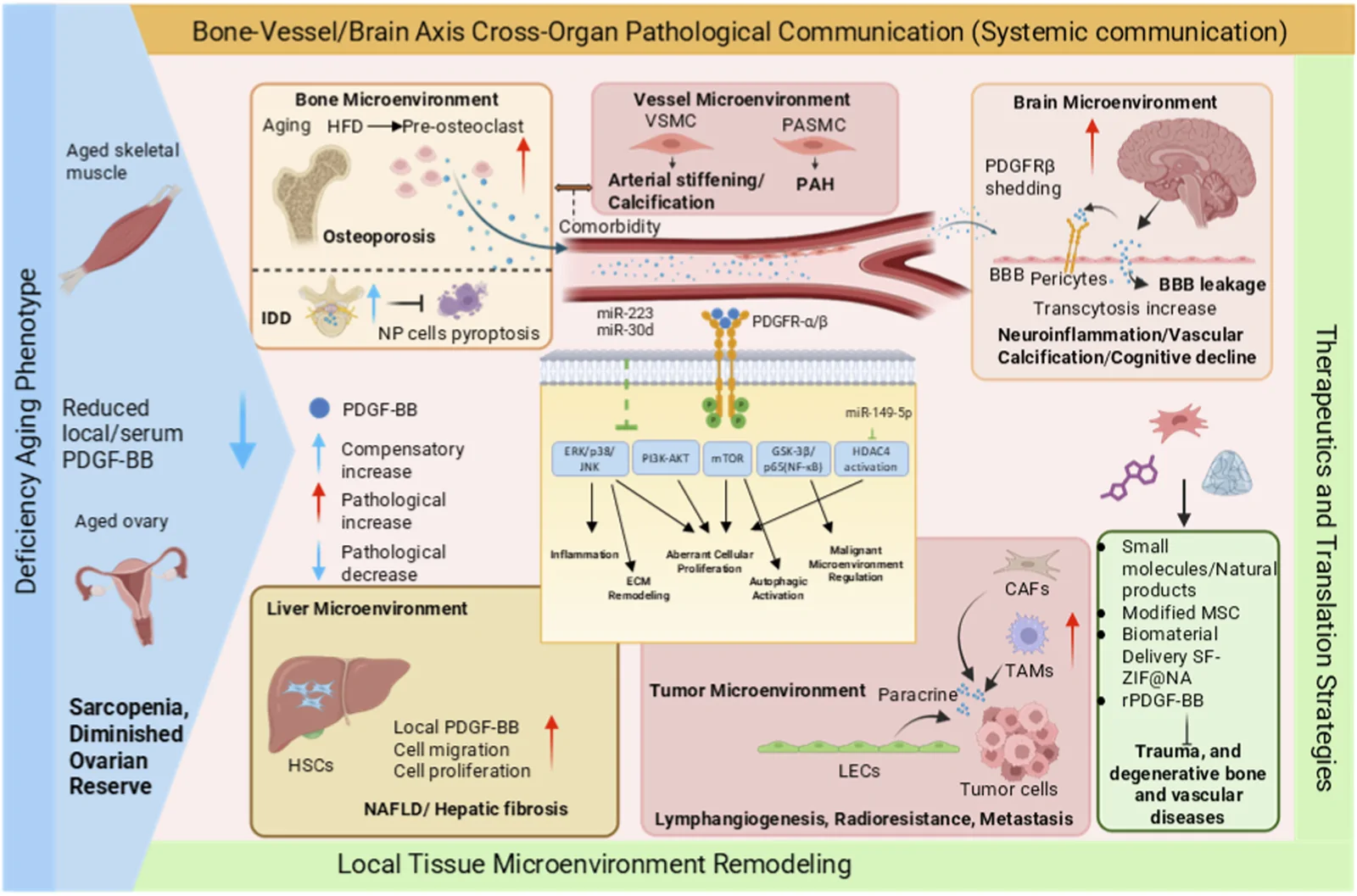
The pleiotropic roles and systemic communication networks of PDGF-BB in aging-related diseases. Pathological PDGF-BB alterations drive profound spatiotemporal microenvironmental remodeling. Locally, upregulated PDGF-BB establishes autocrine/paracrine loops driving VSMC/PASMC phenotypic switching, arterial stiffening, and pulmonary arterial hypertension (PAH), while neuronal PDGF-BB accumulation perturbs brain homeostasis. Systemically, metabolic stress induces massive PDGF-BB spillover from pre-osteoclasts, establishing a pathological bone-vessel-brain axis that couples osteoporosis with vascular calcification and blood-brain barrier (BBB) dysfunction. In other niches, compensatory PDGF-BB limits nucleus pulposus (NP) cell pyroptosis during intervertebral disc degeneration (IDD), whereas its deficiency accelerates sarcopenia and ovarian aging. Furthermore, hyperactivated PDGF-BB accelerates liver fibrosis via hepatic stellate cells (HSCs) and fuels tumor metastasis. Mechanistically, PDGFR-α/β engagement triggers complex intracellular kinase, inflammatory, autophagic, and epigenetic networks, which are endogenously fine-tuned by microRNAs (e.g., miR-223, miR-30 d). Targeting these networks via small molecules, natural products, or smart biomaterials provides promising translational strategies for age-related degenerative diseases.

Therapeutic strategies leveraging natural products and drug repurposing to target PDGF-BB offer inherent safety advantages, rendering them highly suitable for the long-term, mild intervention of chronic aging-related diseases. Conversely, the integration of biological agents with advanced materials science holds immense promise for achieving high-precision, source-specific spatiotemporal modulation.

Guided by current empirical evidence, future research trajectories must transcend isolated observations to construct a multidimensional understanding of PDGF-BB in aging. At the fundamental level, leveraging single-cell multi-omics and spatial transcriptomics will be crucial to deeply decode its source-specific transcriptional regulatory networks and functional heterogeneities. This mechanistic exploration should concurrently elucidate the intricate crosstalk between PDGF-BB, inflammatory cascades, autophagic pathways, and the senescence-associated secretory phenotype (SASP), thereby facilitating the design of rational multi-target strategies. To translate these molecular insights into practice, innovating smart delivery systems integrated with tissue engineering—such as cell-specific promoter-driven hydrogel scaffolds—is imperative to systematically broaden the molecule’s exceptionally narrow therapeutic window. Ultimately, by comprehensively deepening our grasp of PDGF-BB in systemic aging paradigms and inter-organ crosstalk, the field can actively propel multicenter clinical trials aimed at the combinatory intervention of cardiovascular, cerebrovascular, and multi-organ senescence.

In conclusion, the profound convergence of mechanistic elucidation with advanced material-pharmaceutical delivery technologies is poised to translate PDGF-BB-targeted interventions from theoretical concepts into novel, viable clinical regimens for combatting organismal aging and alleviating associated degenerative disorders.

## References

[B1] Aljabban J. Rohr M. Syed S. Khorfan K. Borkowski V. Aljabban H. (2022). Transcriptome changes in stages of non-alcoholic fatty liver disease. World J. Hepatol. 14 (7), 1382–1397. 10.4254/wjh.v14.i7.1382 36158924 PMC9376779

[B2] Andrae J. Gallini R. Betsholtz C. (2008). Role of platelet-derived growth factors in physiology and medicine. Genes Dev. 22 (10), 1276–1312. 10.1101/gad.1653708 18483217 PMC2732412

[B3] Arai T. Hirose M. Kagawa T. Hatsuda K. Inoue Y. (2022). Platelet-derived growth factor can predict survival and acute exacerbation in patients with idiopathic pulmonary fibrosis. J. Thorac. Dis. 14 (2), 278–294. 10.21037/jtd-21-1418 35280478 PMC8902107

[B4] Barrientos S. Stojadinovic O. Golinko M. S. Brem H. Tomic-Canic M. (2008). Growth factors and cytokines in wound healing. Wound Repair Regen. 16 (5), 585–601. 10.1111/j.1524-475X.2008.00410.x 19128254

[B5] Barroeta-Espar I. Weinstock L. D. Perez-Nievas B. G. Meltzer A. C. Siao T. C. M. Amaral A. C. (2019). Distinct cytokine profiles in human brains resilient to Alzheimer's pathology. Neurobiol. Dis. 121, 327–337. 10.1016/j.nbd.2018.10.009 30336198 PMC6437670

[B6] Borkham-Kamphorst E. Weiskirchen R. (2016). The PDGF system and its antagonists in liver fibrosis. Cytokine Growth Factor Rev. 28, 53–61. 10.1016/j.cytogfr.2015.10.002 26547628

[B7] Bouet P. E. Boueilh T. de la Barca J. M. C. Boucret L. Blanchard S. Ferre-L'Hotellier V. (2020). The cytokine profile of follicular fluid changes during ovarian ageing. J. Gynecol. Obstet. Hum. Reprod. 49 (4), 101704. 10.1016/j.jogoh.2020.101704 32028036

[B8] Calvani R. Picca A. Marini F. Biancolillo A. Gervasoni J. Persichilli S. (2021). Identification of biomarkers for physical frailty and sarcopenia through a new multi-marker approach: results from the BIOSPHERE study. Geroscience 43 (2), 727–740. 10.1007/s11357-020-00197-x 32488674 PMC8110636

[B9] Campisi J. (2013). Aging, cellular senescence, and cancer. Annu. Rev. Physiol. 75, 685–705. 10.1146/annurev-physiol-030212-183653 23140366 PMC4166529

[B10] Chen W. Baylink D. J. Brier-Jones J. Neises A. Kiroyan J. B. Rundle C. H. (2015). PDGFB-Based stem cell gene therapy increases bone strength in the mouse. Proc. Natl. Acad. Sci. U. S. A. 112 (29), E3893–E3900. 10.1073/pnas.1501759112 26150503 PMC4517286

[B11] Chen C. C. Li H. Y. Leu Y. L. Chen Y. J. Wang C. J. Wang S. H. (2020). Corylin inhibits vascular cell inflammation, proliferation and migration and reduces atherosclerosis in ApoE-Deficient mice. Antioxidants (Basel) 9 (4), 275. 10.3390/antiox9040275 32218307 PMC7222202

[B12] Chen X. Yu X. Lian G. Tang H. Yan Y. Gao G. (2024). Canagliflozin inhibits PASMCs proliferation via regulating SGLT1/AMPK signaling and attenuates artery remodeling in MCT-Induced pulmonary arterial hypertension. Biomed. Pharmacother. 174, 116505. 10.1016/j.biopha.2024.116505 38574614

[B13] Chen Z. Yang W. Tang Y. Dong Q. Huang K. Tan J. (2025). Trifunctional sialylation-based SF-ZIF@NA hydrogel for selective osteoclast inhibition and enhanced bone-vessel regeneration in osteoporotic bone defects. Adv. Sci. (Weinh) 12 (19), e2415895. 10.1002/advs.202415895 40135851 PMC12097110

[B14] Contreras O. Cordova-Casanova A. Brandan E. (2021). PDGF-PDGFR network differentially regulates the fate, migration, proliferation, and cell cycle progression of myogenic cells. Cell Signal 84, 110036. 10.1016/j.cellsig.2021.110036 33971280

[B15] Contreras J. A. Fujisaki K. Ortega N. E. Barisano G. Sagare A. Pappas I. (2024). Decreased functional connectivity is associated with increased levels of cerebral spinal fluid soluble-PDGFRbeta, a marker of blood brain barrier breakdown, in older adults. Brain Imaging Behav. 18 (6), 1343–1354. 10.1007/s11682-024-00912-8 39254921 PMC11680618

[B16] Czochra P. Klopcic B. Meyer E. Herkel J. Garcia-Lazaro J. F. Thieringer F. (2006). Liver fibrosis induced by hepatic overexpression of PDGF-B in transgenic mice. J. Hepatol. 45 (3), 419–428. 10.1016/j.jhep.2006.04.010 16842882

[B17] Er P. Qian D. Zhang W. Zhang B. Wei H. Zhang T. (2020). The expression of PDGF-BB predicts curative effect in locally advanced esophageal squamous cell carcinoma treated by radiotherapy. Aging (Albany NY) 12 (8), 6586–6599. 10.18632/aging.102993 32330901 PMC7202496

[B18] Evanson J. R. Guyton M. K. Oliver D. L. Hire J. M. Topolski R. L. Zumbrun S. D. (2014). Gender and age differences in growth factor concentrations from platelet-rich plasma in adults. Mil. Med. 179 (7), 799–805. 10.7205/MILMED-D-13-00336 25003868

[B19] Fane M. Weeraratna A. T. (2020). How the ageing microenvironment influences tumour progression. Nat. Rev. Cancer 20 (2), 89–106. 10.1038/s41568-019-0222-9 31836838 PMC7377404

[B20] Fang R. C. Galiano R. D. (2008). A review of becaplermin gel in the treatment of diabetic neuropathic foot ulcers. Biologics 2 (1), 1–12. 10.2147/btt.s1338 19707423 PMC2727777

[B21] Fang J. Huang X. Han X. Zheng Z. Hu C. Chen T. (2020). Endothelial progenitor cells promote viability and nerve regenerative ability of mesenchymal stem cells through PDGF-BB/PDGFR-beta signaling. Aging (Albany NY) 12 (1), 106–121. 10.18632/aging.102604 31899688 PMC6977666

[B22] Feng S. K. Chen T. H. Li H. M. Cao J. Liu D. B. Rao S. S. (2021). Deficiency of Omentin-1 leads to delayed fracture healing through excessive inflammation and reduced CD31(hi)Emcn(hi) vessels. Mol. Cell Endocrinol. 534, 111373. 10.1016/j.mce.2021.111373 34174367

[B23] Franceschi C. Garagnani P. Parini P. Giuliani C. Santoro A. (2018). Inflammaging: a new immune-metabolic viewpoint for age-related diseases. Nat. Rev. Endocrinol. 14 (10), 576–590. 10.1038/s41574-018-0059-4 30046148

[B24] Fredriksson L. Li H. Eriksson U. (2004). The PDGF family: four gene products form five dimeric isoforms. Cytokine Growth Factor Rev. 15 (4), 197–204. 10.1016/j.cytogfr.2004.03.007 15207811

[B25] Gao L. Zhuang Y. Liu Z. (2025). Nogo-B silencing expedites the senescence of platelet-derived growth Factor-BB-Induced human hepatic stellate cells Via autophagy. Mol. Biotechnol. 67 (5), 2023–2034. 10.1007/s12033-024-01179-6 38727882

[B26] Goetzl E. J. Schwartz J. B. Mustapic M. Lobach I. V. Daneman R. Abner E. L. (2017). Altered cargo proteins of human plasma endothelial cell-derived exosomes in atherosclerotic cerebrovascular disease. FASEB J. 31 (8), 3689–3694. 10.1096/fj.201700149 28476896 PMC5503715

[B27] Guerit E. Arts F. Dachy G. Boulouadnine B. Demoulin J. B. (2021). PDGF receptor mutations in human diseases. Cell Mol. Life Sci. 78 (8), 3867–3881. 10.1007/s00018-020-03753-y 33449152 PMC11072557

[B28] Guo X. Zhang H. He C. Qin K. Lai Q. Fang Y. (2024). RUNX1 promotes angiogenesis in colorectal cancer by regulating the crosstalk between tumor cells and tumor associated macrophages. Biomark. Res. 12 (1), 29. 10.1186/s40364-024-00573-1 38419056 PMC10903076

[B29] Ha J. M. Yun S. J. Kim Y. W. Jin S. Y. Lee H. S. Song S. H. (2015). Platelet-derived growth factor regulates vascular smooth muscle phenotype via Mammalian target of rapamycin complex 1. Biochem. Biophys. Res. Commun. 464 (1), 57–62. 10.1016/j.bbrc.2015.05.097 26032503

[B30] Huang J. Li Y. Y. Xia K. Wang Y. Y. Chen C. Y. Chen M. L. (2021). Harmine targets inhibitor of DNA binding-2 and activator protein-1 to promote preosteoclast PDGF-BB production. J. Cell Mol. Med. 25 (12), 5525–5533. 10.1111/jcmm.16562 33960660 PMC8184727

[B31] Ismaeel A. Miserlis D. Papoutsi E. Haynatzki G. Bohannon W. T. Smith R. S. (2022). Endothelial cell-derived pro-fibrotic factors increase TGF-beta1 expression by smooth muscle cells in response to cycles of hypoxia-hyperoxia. Biochim. Biophys. Acta Mol. Basis Dis. 1868 (1), 166278. 10.1016/j.bbadis.2021.166278 34601016 PMC8629962

[B32] Kagami S. Urushihara M. Kitamura A. Kondo S. Hisayama T. Kitamura M. (2004). PDGF-BB enhances alpha1beta1 integrin-mediated activation of the ERK/AP-1 pathway involved in collagen matrix remodeling by rat mesangial cells. J. Cell Physiol. 198 (3), 470–478. 10.1002/jcp.10433 14755552

[B33] Kapoor A. Nation D. A. , and Alzheimer's Disease Neuroimaging Initiative. (2022). Alzheimer's disease neuroimaging I. Platelet-derived growth factor-BB and white matter hyperintensity burden in APOE4 carriers. Cereb. Circ. Cogn. Behav. 3 100131. 10.1016/j.cccb.2022.100131 PMC9286493 35844252

[B34] Kasurinen J. H. Hagstrom J. Kaprio T. Jalkanen S. Salmi M. Bockelman C. (2023). Prognostic values of tissue and serum angiogenic growth factors depend on the phenotypic subtypes of colorectal cancer. Cancers (Basel) 15 (15), 3871. 10.3390/cancers15153871 37568687 PMC10417397

[B35] Kirchmeyer M. Gaigneaux A. Servais F. A. Arslanow A. Casper M. Krawczyk M. (2023). Altered profiles of circulating cytokines in chronic liver diseases (NAFLD/HCC): impact of the PNPLA3I148M risk allele. Hepatol. Commun. 7 (12), e0306. 10.1097/HC9.0000000000000306 38015590 PMC10667005

[B36] Kisseleva T. Brenner D. (2021). Molecular and cellular mechanisms of liver fibrosis and its regression. Nat. Rev. Gastroenterol. Hepatol. 18 (3), 151–166. 10.1038/s41575-020-00372-7 33128017

[B37] Liang X. Zhou J. Wang H. Zhang Z. Yin M. Zhu Y. (2024). miR-30d attenuates pulmonary arterial hypertension via targeting MTDH and PDE5A and modulates the beneficial effect of sildenafil. Adv. Sci. (Weinh) 11 (40), e2407712. 10.1002/advs.202407712 39206778 PMC11516105

[B38] Liu C. Wang C. Yang F. Lu Y. Du P. Hu K. (2022). The conditioned medium from mesenchymal stromal cells pretreated with proinflammatory cytokines promote fibroblasts migration and activation. PLoS One 17 (4), e0265049. 10.1371/journal.pone.0265049 35404961 PMC9000110

[B39] Liu G. Wang J. Wei Z. Fang C. L. Shen K. Qian C. (2023). Elevated PDGF-BB from bone impairs hippocampal vasculature by inducing PDGFRbeta shedding from pericytes. Adv. Sci. (Weinh) 10 (20), e2206938. 10.1002/advs.202206938 37102631 PMC10369301

[B40] Liu G. Shu W. Chen Y. Fu Y. Fang S. Zheng H. (2024). Bone-derived PDGF-BB enhances hippocampal non-specific transcytosis through microglia-endothelial crosstalk in HFD-Induced metabolic syndrome. J. Neuroinflammation 21 (1), 111. 10.1186/s12974-024-03097-5 38685040 PMC11057146

[B41] Lopez-Otin C. Blasco M. A. Partridge L. Serrano M. Kroemer G. (2023). Hallmarks of aging: an expanding universe. Cell 186 (2), 243–278. 10.1016/j.cell.2022.11.001 36599349

[B42] Ma Q. Wang S. Xue H. Ni L. Yuan P. Shen Y. (2025). Targeting Msx2 as a brake in the fusion fate of osteoclasts and an anabolic therapy in pre-clinical models of osteoporosis. Nat. Commun. 16 (1), 7228. 10.1038/s41467-025-61938-0 40764291 PMC12325673

[B43] Marzetti E. Picca A. Marini F. Biancolillo A. Coelho-Junior H. J. Gervasoni J. (2019). Inflammatory signatures in older persons with physical frailty and sarcopenia: the frailty “cytokinome” at its core. Exp. Gerontol. 122, 129–138. 10.1016/j.exger.2019.04.019 31054959

[B44] Masliah E. Mallory M. Alford M. Deteresa R. Saitoh T. (1995). PDGF is associated with neuronal and glial alterations of alzheimer's disease. Neurobiol. Aging 16 (4), 549–556. 10.1016/0197-4580(95)00050-o 8544904

[B45] McCaffrey T. A. Nicholson A. C. Szabo P. E. Weksler M. E. Weksler B. B. (1988). Aging and arteriosclerosis. The increased proliferation of arterial smooth muscle cells isolated from old rats is associated with increased platelet-derived growth factor-like activity. J. Exp. Med. 167 (1), 163–174. 10.1084/jem.167.1.163 3335829 PMC2188811

[B46] Moslehi J. J. (2016). Cardiovascular toxic effects of targeted cancer therapies. N. Engl. J. Med. 375 (15), 1457–1467. 10.1056/NEJMra1100265 27732808

[B47] Ogawa S. Ochi T. Shimada H. Inagaki K. Fujita I. Nii A. (2010). Anti-PDGF-B monoclonal antibody reduces liver fibrosis development. Hepatol. Res. 40 (11), 1128–1141. 10.1111/j.1872-034X.2010.00718.x 20880061

[B48] Picca A. Calvani R. Cesari M. Landi F. Bernabei R. Coelho-Junior H. J. (2020). Biomarkers of physical frailty and sarcopenia: coming up to the place? Int. J. Mol. Sci. 21 (16), 5635. 10.3390/ijms21165635 32781619 PMC7460617

[B49] Payne L. B. Abdelazim H. Hoque M. Barnes A. Mironovova Z. Willi C. E. (2023). A soluble platelet-derived growth factor receptor-beta originates *via* Pre-mRNA splicing in the healthy brain and is upregulated during hypoxia and aging. Biomolecules 13 (4). 711. 10.3390/biom13040711 37189457 PMC10136073

[B50] Perros F. Montani D. Dorfmuller P. Durand-Gasselin I. Tcherakian C. Le Pavec J. (2008). Platelet-derived growth factor expression and function in idiopathic pulmonary arterial hypertension. Am. J. Respir. Crit. Care Med. 178 (1), 81–88. 10.1164/rccm.200707-1037OC 18420966

[B51] Pinzani M. Gesualdo L. Sabbah G. M. Abboud H. E. (1989). Effects of platelet-derived growth factor and other polypeptide mitogens on DNA synthesis and growth of cultured rat liver fat-storing cells. J. Clin. Invest. 84 (6), 1786–1793. 10.1172/JCI114363 2592560 PMC304056

[B52] Pizarro A. Hayer K. Lahens N. F. Hogenesch J. B. (2013). CircaDB: a database of Mammalian circadian gene expression profiles. Nucleic Acids Res. 41 (Database issue), D1009–D1013. 10.1093/nar/gks1161 23180795 PMC3531170

[B53] Pustlauk W. Roch T. Dimitriou I. M. Blázquez Navarro A. Stervbo U. Anft M. (2025). A complex osteoporotic milieu is associated with arterial stiffening and PDGF-BB-mediated calcification of human smooth muscle cells. bioRxiv 2025. 10.1101/2025.10.25.684499

[B54] Rabinovitch M. (2012). Molecular pathogenesis of pulmonary arterial hypertension. J. Clin. Invest. 122 (12), 4306–4313. 10.1172/JCI60658 23202738 PMC3533531

[B55] Raines E. W. Koyama H. Carragher N. O. (2000). The extracellular matrix dynamically regulates smooth muscle cell responsiveness to PDGF. Ann. N. Y. Acad. Sci. 902, 39–51. 10.1111/j.1749-6632.2000.tb06299.x 10865824

[B56] Reif S. Lang A. Lindquist J. N. Yata Y. Gabele E. Scanga A. (2003). The role of focal adhesion kinase-phosphatidylinositol 3-kinase-akt signaling in hepatic stellate cell proliferation and type I collagen expression. J. Biol. Chem. 278 (10), 8083–8090. 10.1074/jbc.M212927200 12502711

[B57] Richeldi L. du Bois R. M. Raghu G. Azuma A. Brown K. K. Costabel U. (2014). Efficacy and safety of nintedanib in idiopathic pulmonary fibrosis. N. Engl. J. Med. 370 (22), 2071–2082. 10.1056/NEJMoa1402584 24836310

[B58] Rovida E. Navari N. Caligiuri A. Dello Sbarba P. Marra F. (2008). ERK5 differentially regulates PDGF-Induced proliferation and migration of hepatic stellate cells. J. Hepatol. 48 (1), 107–115. 10.1016/j.jhep.2007.08.010 17998143

[B59] Sagare A. P. Sweeney M. D. Makshanoff J. Zlokovic B. V. (2015). Shedding of soluble platelet-derived growth factor receptor-beta from human brain pericytes. Neurosci. Lett. 607, 97–101. 10.1016/j.neulet.2015.09.025 26407747 PMC4631673

[B60] Santhanam L. Liu G. Jandu S. Su W. Wodu B. P. Savage W. (2021). Skeleton-secreted PDGF-BB mediates arterial stiffening. J. Clin. Invest. 131 (20), e147116. 10.1172/JCI147116 34437300 PMC8516464

[B61] Selimovic N. Bergh C. H. Andersson B. Sakiniene E. Carlsten H. Rundqvist B. (2009). Growth factors and interleukin-6 across the lung circulation in pulmonary hypertension. Eur. Respir. J. 34 (3), 662–668. 10.1183/09031936.00174908 19324949

[B62] Su F. Shi M. Zhang J. Zheng Q. Wang H. Li X. (2020). MiR-223/NFAT5 signaling suppresses arterial smooth muscle cell proliferation and motility in vitro. Aging (Albany NY) 12 (24), 26188–26198. 10.18632/aging.202395 33373321 PMC7803580

[B63] Su Z. Liu J. Zheng Z. Zhen L. Hu X. Luo D. (2025). Recent advances in the crosstalk between bone and vascular system: from mechanism to therapy. Cell Signal 135, 112001. 10.1016/j.cellsig.2025.112001 40675234

[B64] Tang L. Xia Z. Luo Z. Long H. Zhu Y. Zhao S. (2017). Low plasma PDGF-BB levels are associated with estradiol in postmenopausal osteoporosis: PDGF-BB mediated by estradiol in women. J. Int. Med. Res. 45 (4), 1332–1339. 10.1177/0300060517706630 28606019 PMC5625528

[B65] Tsuchida T. Friedman S. L. (2017). Mechanisms of hepatic stellate cell activation. Nat. Rev. Gastroenterol. Hepatol. 14 (7), 397–411. 10.1038/nrgastro.2017.38 28487545

[B66] Usui T. Morita T. Okada M. Yamawaki H. (2014). Histone deacetylase 4 controls neointimal hyperplasia via stimulating proliferation and migration of vascular smooth muscle cells. Hypertension 63 (2), 397–403. 10.1161/HYPERTENSIONAHA.113.01843 24166750

[B67] Wang M. Jiang L. Monticone R. E. Lakatta E. G. (2014). Proinflammation: the key to arterial aging. Trends Endocrinol. Metab. 25 (2), 72–79. 10.1016/j.tem.2013.10.002 24365513 PMC3917314

[B68] Wang M. Kim S. H. Monticone R. E. Lakatta E. G. (2015). Matrix metalloproteinases promote arterial remodeling in aging, hypertension, and atherosclerosis. Hypertension 65 (4), 698–703. 10.1161/HYPERTENSIONAHA.114.03618 25667214 PMC4359070

[B69] Wang M. Zhang L. Zhu W. Zhang J. Kim S. H. Wang Y. (2018). Calorie restriction curbs proinflammation that accompanies arterial aging, preserving a youthful phenotype. J. Am. Heart Assoc. 7 (18), e009112. 10.1161/JAHA.118.009112 30371211 PMC6222931

[B70] Wang C. Liu Y. He D. (2019). Diverse effects of platelet-derived growth factor-BB on cell signaling pathways. Cytokine 113, 13–20. 10.1016/j.cyto.2018.10.019 30539778

[B71] Wang J. Fang C. L. Noller K. Wei Z. Liu G. Shen K. (2023). Bone-derived PDGF-BB drives brain vascular calcification in Male mice. J. Clin. Invest. 133 (23), e168447. 10.1172/JCI168447 37815871 PMC10688993

[B72] Xie H. Cui Z. Wang L. Xia Z. Hu Y. Xian L. (2014). PDGF-BB secreted by preosteoclasts induces angiogenesis during coupling with osteogenesis. Nat. Med. 20 (11), 1270–1278. 10.1038/nm.3668 25282358 PMC4224644

[B73] Yan J. Xiao G. Yang C. Liu Q. Lv C. Yu X. (2024). Cancer-associated fibroblasts promote lymphatic metastasis in cholangiocarcinoma via the PDGF-BB/PDGFR-beta mediated paracrine signaling network. Aging Dis. 15 (1), 369–389. 10.14336/AD.2023.0420 37307823 PMC10796099

[B74] Yang X. P. Pei Z. H. Ren J. (2009). Making up or breaking up: the tortuous role of platelet-derived growth factor in vascular ageing. Clin. Exp. Pharmacol. Physiol. 36 (8), 739–747. 10.1111/j.1440-1681.2009.05182.x 19473339

[B75] Younan S. A. Ueland T. E. Savitz B. L. Jerome R. N. Budine T. D. Hawkins A. T. (2026). Recombinant platelet-derived growth factor in tissue repair: a review exploring frontiers in regenerative medicine. Plast. Reconstr. Surg. 157 (4), 759–770. 10.1097/PRS.0000000000012426 40952157 PMC13007911

[B76] Yu X. Elfimova N. Muller M. Bachurski D. Koitzsch U. Drebber U. (2022). Autophagy-related activation of hepatic stellate cells reduces cellular miR-29a by promoting its vesicular secretion. Cell Mol. Gastroenterol. Hepatol. 13 (6), 1701–1716. 10.1016/j.jcmgh.2022.02.013 35219894 PMC9046234

[B77] Zaidi M. Lizneva D. Yuen T. (2021). The role of PDGF-BB in the bone-vascular relationship during aging. J. Clin. Invest. 131 (20), e153644. 10.1172/JCI153644 34651590 PMC8516459

[B78] Zhan Y. Kim S. Izumi Y. Izumiya Y. Nakao T. Miyazaki H. (2003). Role of JNK, p38, and ERK in platelet-derived growth factor-induced vascular proliferation, migration, and gene expression. Arterioscler. Thromb. Vasc. Biol. 23 (5), 795–801. 10.1161/01.ATV.0000066132.32063.F2 12637337

[B79] Zhang J. M. Feng F. E. Wang Q. M. Zhu X. L. Fu H. X. Xu L. P. (2016). Platelet-derived growth Factor-BB protects mesenchymal stem cells (MSCs) derived from immune thrombocytopenia patients against apoptosis and senescence and maintains MSC-mediated immunosuppression. Stem Cells Transl. Med. 5 (12), 1631–1643. 10.5966/sctm.2015-0360 27471307 PMC5189646

[B80] Zhang B. Dong Y. Liu M. Yang L. Zhao Z. (2019). miR-149-5p inhibits vascular smooth muscle cells proliferation, invasion, and migration by targeting histone deacetylase 4 (HDAC4). Med. Sci. Monit. 25, 7581–7590. 10.12659/MSM.916522 31595884 PMC6796703

[B81] Zhang W. Gong Y. Zheng X. Qiu J. Jiang T. Chen L. (2021). Platelet-derived growth Factor-BB inhibits intervertebral disc degeneration via suppressing pyroptosis and activating the MAPK signaling pathway. Front. Pharmacol. 12, 799130. 10.3389/fphar.2021.799130 35095507 PMC8795915

[B82] Zhang C. Diaz-Hernandez M. E. Fukunaga T. Shenoy S. Yoon S. T. Haglund L. (2025). Therapeutic effects of PDGF-AB/BB against cellular senescence in human intervertebral disc. Elife 13, RP103073. 10.7554/eLife.103073 40668091 PMC12266719

[B83] Zheng X. Qiu J. Ye J. Gong Y. Jiang T. Gao N. (2024). Macrophage-derived PDGF-BB modulates glycolytic enzymes expression and pyroptosis in nucleus pulposus cells via PDGFR-beta/TXNIP pathway. Osteoarthr. Cartil. 32 (10), 1245–1260. 10.1016/j.joca.2024.05.001 38744373

